# The Role of Psychological Inflexibility and Experiential Approach on Mental Health in Children and Adolescents: An Exploratory Study

**DOI:** 10.3390/bs12070201

**Published:** 2022-06-22

**Authors:** Gloria Torres-Fernández, Miguel Rodríguez-Valverde, Salvador Reyes-Martín, Mónica Hernández-Lopez

**Affiliations:** Department of Psychology, University of Jaén, Campus Las Lagunillas s/n, 23071 Jaén, Spain; gmtf0001@red.ujaen.es (G.T.-F.); srm00046@red.ujaen.es (S.R.-M.); mhlopez@ujaen.es (M.H.-L.)

**Keywords:** psychological inflexibility, experiential approach, emotional intelligence, mental health, children

## Abstract

The prevalence of mental health problems during childhood and adolescence is on the rise. There is a growing interest in the examination of personal variables that may function as risk factors and that may be targeted for effective intervention. This study explores the relationships amongst different aspects of psychological inflexibility (one, typically studied, focusing on the individual’s responding to unwanted emotions and cognitions, and another, more recently explored, focusing on the individual’s responding to desired thoughts and affective states), emotional intelligence, and mental health symptoms. A total of 129 school-going children (mean age: 11.16 years old) completed a battery of instruments comprising the Avoidance and Fusion Questionnaire-Youth (AFQ-Y17), the Experiential Approach Scale (EAS), the Emotional Intelligence Quotient Inventory (EQi-YV), and the Revised Child Anxiety and Depression Scale (RCADS-30). Results showed that both the AFQ-Y17 score and an EAS subscale score (Anxious Clinging) were significant independent predictors of mental health symptoms in general. Emotional intelligence was predictive only for depression, and both the AFQ-Y17 and the Anxious Clinging EAS subscale significantly incremented the predictive power of a hierarchical linear regression model including all three variables. These results underscore the relevance of psychological inflexibility for child/adolescent mental health, and the need to further explore a specific aspect of inflexibility regarding positive emotions and other appetitive private events.

## 1. Introduction

The prevalence of mental health problems in children and adolescents has been on the rise for the last few decades [1,2]. This constitutes a major public health concern, not only because of the suffering it presently inflicts on children and adolescents and their families, but also because the available evidence points out that a substantial proportion of psychological disorders diagnosed during childhood and adolescence will not remit and will persist well into adulthood [3,4]. The COVID-19 pandemic has only worsened things, and an increasing number of voices are warning about the serious consequences this will have for generations to come [5].

An ample literature exists on research that examines risk and protection factors for the development of psychological disorders during childhood and adolescence. While social and family factors are credited with an essential role in the development of these problems [6,7], there is a growing interest in the examination of personal tendencies and coping styles that might make children and adolescents more or less vulnerable to the effects of environmental stressors [8]. A better understanding of these personal risk factors, especially those that are malleable and thus likely to be modified through effective psychological interventions, might place us in a better position to adequately address this serious mental health concern.

### 1.1. Emotional Intelligence and Psychopathology

Since the 1990s, emotional intelligence (EI) has gathered growing research interest, with particular attention paid to its relationship with mental health. Over the years, different definitions and models of EI have appeared. Most of them share in common that they describe some aspect of personality, and to some degree, understand EI as a trait, or as having trait-like qualities [9]. One of the most popular models of EI is Bar-On’s [10], which defines EI as: “an array of non-cognitive capabilities, competencies, and skills that influence one’s ability to succeed in coping with environmental demands and pressures” [11] (p. 14). The Emotional Quotient Inventory (EQi), derived from this model, is one of the most frequently used measures of trait EI. Regardless of the different models and approaches to the construct, EI appears to be a relevant construct for mental health. An extensive literature has collected evidence on the relationship between poor EI and psychopathology. Specifically, research with adults has shown a negative association between EI and depression [12,13,14] and anxiety disorders [15]. Research with children and adolescents has also examined how mental health is associated with EI [16]. EI has been shown to correlate negatively with depression [17,18] and anxiety [19,20].

### 1.2. Psychological Inflexibility and Mental Health

A different approach to how individuals perceive and react to their emotions has developed from the functional-contextual perspective of Acceptance and Commitment Therapy (ACT) [21,22]. ACT is arguably the most representative and empirically supported of the Third Wave of cognitive-behavioral therapies [23]. ACT focuses on how individuals respond to their own behavior, specifically to their private events (e.g., thoughts, emotions, bodily sensations), and how this response can facilitate or interfere with leading a valued life. The key construct that has emerged from this perspective is that of psychological flexibility (PF). PF involves being open to experiencing private events in the present moment as a conscious human being, persisting or changing in behavior in response to situational demands in pursuit of personally valued directions [24]. PF is developed through the individual’s interaction with their social-verbal context, as they learn to discriminate and respond to their own behavior, and particularly to their private events. PF is conceptualized as a learned generalized pattern of responding to one’s private events that, although stable to some extent (and measurable with psychometric instruments [25]) is context-dependent, malleable, and thus can be enhanced through intervention. Indeed, ACT aims to increase PF by teaching clients to be more open to their private experience as it occurs (rather than attempting to control it) in the service of living a life driven by personally chosen values [22].

ACT conceptualizes most psychological disorders as resulting from extended, generalized patterns of responding to one’s private events characterized by a lack of flexibility (or presence of inflexibility). Psychological inflexibility (PI) is characterized by cognitive fusion (i.e., the excessive control that the verbal functions of private events exert over the individual’s behavior, particularly literality in following self-referential, evaluative rules) and ensuing inflexible patterns of experiential avoidance (unwillingness to contact with one’s own private aversive experience and persistent avoidance of unwanted cognitions and emotions and their situational triggers) [26,27]. While experiential avoidance may provide an immediate relief, in the mid/long run it will bring about more distress and an exacerbation of psychological symptoms, since it frequently interferes with the individual’s engagement in valued actions [26]. In order to cope with this further distress, the individual might engage in further avoidance behavior, entering a vicious cycle of entrapment in behavior patterns that are inconsistent with a valued life, but nonetheless maintained by negative reinforcement (the short-lived, immediate relief from distress). PI is considered a transdiagnostic dimension, inasmuch as it can functionally underlie many formally different psychological disorders [28]. The very ample research with adult samples has shown that PI strongly predicts emotional distress, psychopathology, and poor mental health and quality of life [28,29,30,31,32,33]. Research with children and adolescents is less abundant, but nonetheless, the available evidence shows that PI is associated with both externalizing and internalizing mental health problems, particularly with symptoms of anxiety and mood disorders [34,35,36,37,38].

### 1.3. Experiential Approach: Responding to Appetitive Private Events

The vast majority of existing research on PF/PI has focused on responding to aversive private events (i.e., how individuals respond to unwanted cognitions and affective states). However, there is nothing in the PF model that necessarily leads to constraining the concept of psychological (in)flexibility to aversive experiences. Arguably, deliberate attempts at controlling positive cognitions and emotions (e.g., desired affective states) might as well be problematic if they become generalized, inflexible patterns of responding that end up interfering with valued living. Recently, Swails et al. [39] presented the construct of experiential approach (EA), which can be characterized as “involving attempts to contact, sustain, or somehow control positive thoughts, emotions, urges, memories, and bodily sensations, as well as the contexts that give rise to them” [39] (p. 528). Accordingly, EA could be thought of as a dimension complementary to experiential avoidance, although thus far it has received considerably less research attention. EA would be problematic insofar as it compromises most of the individual’s resources, interfering with personally valued actions that might not feel pleasant in the short run but would lead to longer-lasting life satisfaction and happiness [6]. To this date, research on EA as a potentially relevant facet of PI is very limited, with only two studies conducted with adult samples. Swails et al. [39] developed the Experiential Approach Scale (EAS), a self-report instrument for the assessment of EA that comprises two factors reflecting rather different dimensions of EA (and that can be argued to constitute two independent subscales representing different forms of relating to appetitive private events). On the one hand, Anxious Clinging (AC) is a tendency to capture and hold on to desired, pleasant emotions, dreading that they will disappear (rather than just savoring them while they last). On the other, Experience Prolonging (EP) refers to simply appreciating every joyful moment for as long as it lasts, without deliberate attempts to holding onto it. This factor structure was confirmed in an adaptation of the EAS for the Spanish population [40]. The available evidence [39,40] shows that AC is strongly positively correlated with experiential avoidance. Likewise, it correlates positively (at least moderately) with different measures of distress and dysfunction (negative affect, neuroticism, anxiety, and depression), while it is negatively correlated with positively valenced variables like subjective happiness and satisfaction with life. The role of EP is less clear. Swails et al. [39] found modest positive correlations between this subscale and experiential avoidance, and very weak or insignificant correlations with mental health criterion variables. Reyes-Martín et al. [40] did not find significant correlations between EP and experiential avoidance, but found the former to correlate positively with positively valenced variables (life satisfaction, subjective happiness, and positive affect) and negatively with negative affect and psychopathology. Accordingly, this emerging literature points to AC as a predictor of suffering and a risk factor for mental health (just like experiential avoidance and PI more generally), while EP could be seen as inert, or even as a protective factor. It is worth noting that while these studies showed that AC and experiential avoidance were correlated, and each of them was in turn correlated with psychopathology and distress, neither explored their relative contribution to these criterion variables.

So far, thus, no study has explored the relative contributions of different aspects of PI regarding the types of private events that are experienced (i.e., aversive vs. appetitive) with children and adolescents (for the sake of clarity, in what follows we will use the term PI to refer to inflexible patterns of responding regarding aversive private events, i.e., unwanted cognitions and emotions, while we will use EA and its subcomponents AC and EP to refer to responding to appetitive private events, i.e., desired affective states and cognitions; however, we conceive EA as an aspect of PI in general). This is an important issue both practically (for a more accurate identification of potential processes that can be targeted for intervention) and theoretically (for a more profound examination of conceptual aspects regarding the psychological flexibility model). The goal of the present study was to explore the extent to which PI and EA are associated with and predict relevant mental health symptoms (anxiety and depression), compared to a traditional trait-like measure of EI.

## 2. Materials and Methods

### 2.1. Participants

A convenience sample of 129 primary and secondary school students with ages ranging between 9.33 and 14.83 years old (M = 11.16; SD = 1.32; 45% female) was recruited from a chartered school in a mid-sized town in Southern Spain. All of them showed typical development and normal reading abilities for their age range (as reported by their teachers). Participants did not receive any incentive for participation, financial or otherwise. Informed consent was obtained from participants and their legal guardians before conducting the study.

This sample size allowed for a minimum statistical power (1-β) of 0.80 for medium effect sizes for all statistical tests performed in the data analyses, as calculated with G*Power software (version 3.1; Heinrich Heine Universität, Düsseldorf).

### 2.2. Instruments

Avoidance and Fusion Questionnaire–Youth (AFQ-Y17 [35], Spanish adaptation and validation [38]). The AFQ-Y17 is a 17-item self-report questionnaire that measures PI in children and adolescents. It is answered on a 0–4 Likert scale (0 = not true; 4 = very true). Items reflect a dominance of the verbal functions of private events (cognitive fusion) that leads to the persistent avoidance of such events and of their situational triggers (experiential avoidance). Example items include “I stop doing things that are important to me whenever I feel bad” or “I push away thoughts and feelings that I don’t like”. High scores are indicative of high levels of PI (and low levels of flexibility). It has a two-factor structure, with a component of Experiential Avoidance and a component of Cognitive Fusion. Both processes are distinct but highly interrelated, and the total score of the measure (ranging 0–68) can be used as a general index of PI. The Spanish adaptation has good internal consistency (α = 0.87) and predictive validity.

Experiential Approach Scale (EAS [39]; Spanish adaptation [40]). The EAS is a self-report questionnaire for the assessment of EA that has been validated with adults (college students). It comprises 18 items that are answered on a 7-point Likert-type scale (1: Never true, to 7: Always true). Like the original instrument, the Spanish adaptation comprises two subscales: Anxious Clinging (AC) comprises 12 items (e.g., “When I’m in a good mood, I worry that something will spoil it” and “When I am having fun, I feel that the experience will not last”), and Experience Prolonging (EP) comprises the other 6 items (e.g., “I try to hang on to feelings I enjoy” and “When I’m feeling good, I try to do whatever I can to hang on to it”). Higher scores reveal stronger effort to control and maintain positive private experiences. The Spanish version that we used presents good internal consistency values (Cronbach’s α = 0.85 for the total scale; αs = 0.89 and 0.90 for the AC and EP subscales, respectively).

Emotional Quotient Inventory: Young Version (EQ-i YV [41]; Spanish validation [42]). The EQ-i YV is a measure of EI for children and adolescents between 6 and 18 years of age. It comprises 60 items that are answered on a four-point scale (1 = never happens to me; 4 = always happens to me). It assesses five areas: Intrapersonal, Interpersonal, Adaptability, Stress management, and General Mood. It includes two validity scales: Positive image scale and Inconsistency index. It shows adequate reliability for each of its dimensions (αs between 0.63 and 0.80) [42]. High scores on the total scale are indicative of good social and adaptive functioning.

The Revised Child Anxiety and Depression Scale (RCADS-30 [43]). The RCDS-30 is a 30-item self-report questionnaire answered on a 4-point Likert (0 = Never; 3 = Always). It comprises 6 subscales (major depressive disorder, panic disorder, social phobia, separation anxiety disorder, generalized anxiety disorder, and obsessive compulsive disorder). The RCADS-30 correlates strongly with other measures of depression, negative affect, fear, and anxiety. Higher scores on the scale are indicative of the probable presence of depression or anxiety disorders. The Spanish adaptation of the instrument presents good internal consistency values (αs between 0.68 and 0.78 for the subscales, and 0.89 for the total scale).

### 2.3. Procedure

The study was conducted at the participants’ school. Study information and consent forms were distributed to all parents or legal guardians in compliance with standing regulations and ethical guidelines from the authors’ university Institutional Review Board (IRB), and the study was approved both by the IRB and by the school management team. All participants took part in a larger research project that involved responding to latency-based tests in different conditions (data not included in the present report). Questionnaire administration was conducted previously to participation in the experimental tasks in all cases. Participants completed the questionnaires in one session at their usual classroom during a regular class, supervised both by a research psychologist and the student’s usual teacher. Questionnaires were presented in the following order: AFQ-Y17, EAS, RCADS-30, EQ-iYV. Completion took around 30 min.

### 2.4. Psychometric and Statistical Analysis

This study attempted to examine the relationship amongst different aspects of psychological inflexibility (involved in responding either to unwanted or to desired affective states and cognitions), emotional intelligence, and mental health symptomatology in children and adolescents. Since we were exploring the use of the EAS (which so far has only been tested with young adults) [40], before conducting any correlational or regression analyses amongst different measures, we conducted an exploratory factor analysis (EFA) on the EAS in order to see if the factorial structure based on child/adolescent scores was similar to the one observed with adults. We used Factor 11.05 [44] for factor analysis, and SPSS 20.0 for all other analyses. Sampling adequacy for factor analysis was assessed through the calculation of Kaiser–Meyer–Olkin (KMO) index and Bartlett’s sphericity test. Optimal implementation of parallel minimum rank factor analysis (MRFA) [45] was used in order to determine the number of extracted factors to retain in EFA, with factor retention based on sample eigenvalues greater than the 95 percentile eigenvalues of simulated datasets [45]. In order to examine the EAS factor structure and its goodness of fit with the number of factors retained (based on parallel MRFA), we conducted an exploratory robust unweighted least squares (RULS) factor analysis with robust Promin rotation based on polychoric correlations, with the following indicators of goodness of fit: RMSEA (root mean square error of approximation), NNFI (non-normed fit index), CFI (comparative fit index), and GFI (goodness-of-fit index). Good model fit was defined by values > 0.95 for NNFI, CFI and GFI, and <0.06 for RMSEA [46,47].

Cronbach’s α for the different instruments was calculated with Hayes and Coutts’s OMEGA macro for SPSS [48] as a measure of internal consistency.

We examined associations amongst PI, EA, EI, and mental health symptoms through Pearson product-to-moment correlations. In order to explore how different aspects of PI regarding aversive or appetitive private events could be used to make clinically relevant predictions regarding mental health symptoms, we conducted hierarchical regression analyses with general mental health symptomatology (total RCADS score) and specific symptoms (subscale RCADS scores) as the criterion variables. For each analysis, we compared a model in which the criterion variable was predicted by PI regarding aversive private events (AFQ-Y17 scores) with a model that also included as a predictor an aspect of PI regarding appetitive private events (the AC subscale of the EAS), in order to see whether and how these different aspects increased the predictive power of the model. EI was included as a predictor in the model, in case it correlated significantly with the criterion variable.

## 3. Results

### 3.1. Factor Structure of the EAS

Since, thus far, the EAS has been used only with adult samples [39,40], we first conducted an EFA in order to explore its factor structure with our child/adolescent sample. The fair KMO index (0.711) and Bartlett’s sphericity test [χ^2^_(153)_ = 890.6; *p* ≤ 0.001] were indicative of the sample adequacy for factor analysis. Two factors were retained (see Table 1) based on the parallel MRFA.

Factor loadings for the different EAS items (see [40] for item description) according to the RULS factor analysis are presented in Table 2. The different items loaded onto the same factors as with adults (using the same Spanish version of the measure [40]): the first factor comprised the twelve items from the AC subscale (items: 1, 2, 3, 5, 7, 8, 13, 14, 15, 16, 17 and 18), accounting for 24.51% of the variance, and the second factor comprised all 6 EP subscale items (items: 4, 6, 9, 10, 11, 12), accounting for 18.90% of the variance. There were no items with absolute factor loadings >0.32 on both factors (i.e., no relevant cross-loadings), and no items had to be removed for low loading (<0.32) in one factor. This two-factor solution accounted for 43.41% of the variance. Inter-factor correlation was almost insignificant (*r* = 0.014), which points to two independent EAS subscales with the same pattern of items’ factor loadings as in the Spanish adaptation with adults. The different indicators revealed a very good fit for the model: RMSEA = 0.0028; NNFI = 0.988; CFI = 0.991; GFI = 0.954.

The descriptive statistics for each EAS item, the total scale and its subscales are presented in Table 3. For specific items, mean scores ranged from 2.28 (Item 18) to 6.26 (Item 6), and the standard deviations ranged between 1.30 (Item 4) and 2.52 (Item 16). Skewness and kurtosis were lower than 1 for the complete scale and for the AC subscale, but not for the EP subscale. Specifically, items 4, 6, 9, 10, and 12 (all of them belonging to the EP subscale) had skewness and kurtosis values larger than acceptable (>|1.5|). This is indicative that these items may not be appropriate for use with this child/adolescent sample. Accordingly, in all subsequent correlational and regression analyses we only used the AC subscale of the EAS, whose items all had acceptable values. Retaining only the AC subscale seems justified not only on the basis of the psychometric properties of the items, but is also consistent with findings in the extant literature on the EAS with adults [39,40]. So far, research with the EAS has shown that only the AC subscale appears to have a clear role, with consistent significant associations with other aspects of PI (i.e., experiential avoidance), negative affect, and poor mental health. The role of EP is less clear, with some findings indicating that it may be an inert factor in terms of its association with PI and distress.

### 3.2. Descriptives and Internal Consistencies

Table 4 presents the descriptives and internal consistencies for the different measures of PI, EA (anxious clinging), EI, and mental health symptoms. There were no significant differences between girls and boys in any of the measures, with the Social Phobia subscale of the RCADS-30 being the one that more closely approached a significant difference (t (127) = 1.690, *p* = 0.094). Internal consistency was good for all the scales, with αs ranging from 0.80 (EAS*_Anxious Clinging_*) to 0.91 (EQi-YV). It was acceptable/good for the RCADS-30 subscales (αs ranging from 0.65 to 0.76) but the Obsessive Compulsive subscale (α = 0.53). Item deletion did not increase reliability for any of the measures or its subscales.

### 3.3. Relationships Amongst Psychological Inflexibility, Experiential Approach, Emotional Intelligence, and Mental Health Symptoms

Table 5 presents correlations amongst PI (AFQ-Y17), the AC dimension of EA (EAS*_Anxious Clinging_*), EI (EQi-YV), and mental health symptoms (RCADS-30). As expected, there was a moderate positive correlation between PI and AC. Both PI and AC showed strong positive correlations of similar magnitude with the total RCADS-30 score (mental health symptoms), and a similar pattern of significant positive correlations with the different RCADS-30 subscales, ranging from 0.251 (AC and separation anxiety) to 0.50 (AC and panic disorder). EI was not significantly associated with total scores of mental health symptoms, nor with any of the anxiety subscales. However, there was a significant negative correlation between EI and major depression, with an absolute magnitude similar to the positive correlations of major depression with PI and with AC. This pattern of correlations suggests that both PI and AC might be potential predictors of worse mental health, while EI would only be negatively associated with depression.

### 3.4. Predictive Regression Analysis

In order to determine the relative contribution of PI (as measured by the AFQ-Y17) and the AC facet of EA in accounting for variation in mental health symptoms, we conducted a series of hierarchical linear regression analyses with the RCADS-30 total score and all RCADS subscale scores as criterion variables. In each model, AFQ-Y17 score was introduced first as a predictor, then the AC score was introduced in the second step. For the major depression subscale, the analysis included another predictor, EI (which had the largest absolute correlation with this subscale). In this case, EQi-YV score was introduced as predictor in the first step, AFQ-Y17 score in the second step, and the AC score in the third step. Table 6 presents coefficients, statistics, and accounted-for variance for all 7 models. In all cases, PI was a significant predictor of the outcome variable, with the largest effect for the total RCADS-30 score (R^2^ = 0.279) and the smallest for the generalized anxiety subscale (R^2^ = 0.073). The inclusion of AC as a predictor significantly increased accounted-for variation in all cases but one (the separation anxiety subscale). The significant increment in explained variance ranged from 2.8% (for the obsessive compulsive disorder subscale) to 14.2% (for the panic disorder subscale). Besides, in all of these cases (with the exception of obsessive compulsive disorder), AC was a stronger predictor (larger absolute β values) than the AFQ-Y17 score. In the specific case of major depression, which also included EQi-YV score as a predictor, both the inclusion of AFQ-Y17 and AC scores resulted in successive significant increments in accounted-for-variance (the final model accounting for more than twice the variance of the initial model with EI as the only predictor), with the EQi-YV being the strongest (negative) predictor followed by AC.

## 4. Discussion

This study attempted to examine whether, and how, different aspects of inflexibility regarding private events (both aversive and appetitive) were associated with anxiety and depression symptoms in children and adolescents and, more specifically, the relative contribution of these aspects in accounting for variation in these symptoms. To that end, we used a typical PI measure based on responding to aversive private events, a recent measure of EA focused on responding to appetitive private events, and a general, trait-like measure of EI, since EI is a typical variable in the study of emotion regulation and its impact on mental health.

Since the EAS is a relatively unexplored measure and, to date, it has never been used with children and adolescents, we examined its psychometric properties before conducting any correlational or regression analyses. Prior research with this measure [39,40] showed that it has a two-factor structure, wherein only one of them (the AC subscale) correlated strongly with other aspects of PI like experiential avoidance, as well as predicted negatively valenced psychological outcomes (measures of distress and dysfunction). It was important, therefore, to explore whether the same factor structure held and the questionnaire was suitable to be used in a similar fashion as with adults. Results showed that, with this sample, the EAS maintained the same two-factor structure as with adults, but this structure accounted for substantially less variance [40]. In addition, while all of the AC subscale items had acceptable skewness and kurtosis, five of the six items in the EP subscale had larger-than-acceptable values. Hence, we limited our examination of the role of EA as a potential predictor of mental health to the AC subscale of the EAS, which showed good psychometric properties. Since only the AC facet of EA has been proven to have a relevant role in terms of its association with experiential avoidance and mental health symptoms [39,40], it seemed appropriate to explore this facet of EA with children and adolescents.

The analysis of the descriptives showed that the mean scores for EI and anxiety and depression symptoms were consistent with prior research. The average RCADS-30 total score was very close to that from a recent validation of the scale with a non-clinical sample of similarly aged children (*M* = 31.61) [49]. Likewise, the average EQi-YV score was very close to the average in the original Spanish validation (*M* = 113.12) [42]. However, for the AFQ-Y17, the mean score for our sample was substantially lower than the mean in the original Spanish validation (*M =* 47.09) [38]. A possible explanation for this difference is that our sample was younger than the sample in the original validation study. A recent validation of the brief format version of the questionnaire (the AFQ-Y8) [34] found that young children score lower than adolescents. This is compatible with the existing evidence that the frequency and intensity of negative emotions increase with age during adolescence. The literature on normative tendencies in socio-emotional development has shown that at a young age, children experience more positive emotions than at an older age, and that the frequency of experienced positive emotions decreases with age while the frequency of experienced negative emotions increases (peaking in late adolescence/early adulthood) [50]. There is also a consistent pattern regarding the intensity of experienced emotions, with both children and adolescents experiencing more high-intensity emotions (both positive and negative) and less low-intensity emotions than adults [50]. Since the AFQ-Y17 focuses on how children respond to aversive private events, it seems reasonable that scores will be higher at an older age. The aforementioned developmental trends are also compatible with our findings regarding the EAS AC subscale. The average score for this subscale in this study was higher than with adults [40]. This seems reasonable, since positive emotions appear to be more frequent and intense during childhood/early adolescence. It only makes sense, in our view, that difficulties with clinging to positive emotions and their situational triggers play a relevant role in the development of inflexible patterns of responding to experienced private events. We believe that research on PI, thus, should not neglect how children respond to their experience of positive emotions and desired affective states, since this might constitute an aspect of general psychological (in)flexibility at least as relevant as responding to aversive private events. The correlational and regression analyses seem to support this view.

The observed pattern of correlations amongst the EQi-YV, the AFQ-Y17, the EAS AC subscale, and the RCADS-30 (and subscales) yields some interesting results. EI, as measured with the EQi-YV, was generally not related to the other measures. Regarding PI and AC, this measure of EI, based on a model with a strong trait-like component, did not appear to converge with specific measures targeting the more context-dependent, learned responses to private events. Regarding mental health symptoms, EI presented a moderate to strong negative correlation with depression, but not with any of the anxiety subscales or with the RCADS-30 total scale. This is unlike previous research with children that found an association between EI (as measured with the EQi-YV) and anxiety [19,20]. The EQi-YV is a complex trait-like measure comprising a broad variety of items, some of which specifically focus on positive/negative mood (e.g., I am happy, I am not happy) but not on arousal, fear, or worry, which would explain the association with depression, but not with different anxiety disorders. Perhaps different measures of EI based on models more focused on explaining EI in terms of behavior patterns that are effective in reducing unnecessary suffering [51] would be more strongly associated with different aspects of PI and mental health.

The pattern of associations amongst the different aspects of inflexibility regarding private events was theoretically coherent and very similar to the findings from the only two prior studies on EA with adults [39,40]. The correlation between the AFQ-Y17 and the EAS AC subscale was significant but not very strong, which points to the relative independence of these two facets of responding to private events dependent on their being aversive or appetitive. This view is strengthened when we consider the correlations of PI (as measured with the AFQ-Y17) and AC with the RCADS-30 and its subscales. Both PI and AC correlated positively (and with a similar magnitude) with the RCADS-30 total scale and all subscales. Previous studies with children and adolescents that have employed the AFQ-Y17 found similar results in regard to anxiety and depression symptoms [34,38], but to date, no study has considered the potential influence of inflexibility regarding appetitive private events on children’s mental health. Our findings point to AC as a potentially relevant factor in this regard. This idea is further supported by the results of regression analyses. These confirmed that both PI and AC independently and significantly contributed to accounting for variation in mental health symptoms, with AC adding a similar amount of accounted-for-variance to the models as PI. This was the case even for depression, wherein trait EI was already the stronger predictor of the criterion variable.

This study has some limitations that need to be mentioned and that lead us to be cautious in considering its findings. First, it examined only a relatively small convenience sample of schoolchildren from a single school. Future studies might attempt to explore the differential contributions of psychological inflexibility regarding aversive and appetitive private events with more representative non-clinical samples, and also with clinical samples whose emotional experience differs from the normative socio-emotional developmental pattern (i.e., with children who experience more frequent high-intensity negative emotions). Second, the measure of EA that we used is the only available measure so far for this construct, but it has only been tested with adults. Our preliminary findings show that EA (and more specifically, AC) appears to play a relevant role in predicting anxiety and depression symptoms, which would justify further research on the specific adaptation of the EAS (or the creation of an altogether new instrument for the assessment of EA) for children/adolescents, with larger samples and proper psychometric guarantees. Third, we used a typical measure of global EI with a strong trait-like component. As mentioned above, it is possible that using a different measure of EI, more focused on aspects of emotion regulation, would yield a different pattern of relationships with PI, EA, and anxiety and depression symptoms. Fourth, the administration of the different instruments was not counterbalanced. Future studies like those suggested above (with larger, more representative samples, and a specific instrument for the assessment of EA with children) might control for potential effects of presentation order.

In sum, the results of the present study suggest that specific and malleable constructs targeting the individual’s response to their private events, such as PI and the AC dimension of EA, constitute relevant predictors of mental health symptoms. Particularly, they underscore the relevance of considering how flexibly children respond to positive emotions and other appetitive private events, and not only to aversive private events. A growing literature is providing evidence about the role of PI as a risk factor for mental health problems during childhood and adolescence, and the potential beneficial effects of interventions aimed at enhancing PF, such as ACT. However, the focus of existing PI measures and associated interventions is fundamentally on how the individual relates to aversive private events. While this seems logical in the context of intervention with clinical patients who are suffering and thus frequently undergoing aversive experiences, perhaps a focus on how flexibly children respond to appetitive private events would be relevant for prevention. Future studies might undertake a deeper examination of these aspects of PI as potential targets for preventative interventions.

## Figures and Tables

**Table 1:** Parallel analysis—minimum rank factor analysis (MRFA) results.

Factor	Real-Data % of Variance	Mean of Random % of Variance	95 Percentile of Random % of Variance
1	27.20	12.10	13.53
2 *	21.42	10.85	11.82
3	8.61	9.85	10.75
4	7.00	9.01	9.72
5	5.87	8.24	8.86
6	5.08	7.54	8.13
7	4.45	6.90	7.43
8	4.03	6.29	6.83
9	3.42	5.65	6.15
10	2.97	5.04	5.57
11	2.53	4.43	4.99
12	2.42	3.86	4.43
13	1.88	3.28	3.83
14	1.27	2.69	3.32
15	0.97	2.06	2.70
16	0.55	1.43	2.10
17	0.30	0.76	1.40

* Advised number of dimensions when 95 percentile is considered.

**Table 2:** Factor loadings for the EAS items.

EAS Item	Factor 1 (Anxious Clinging)	Factor 2 (Experience Prolonging)
1.	**0.67**	0.11
2.	**0.51**	0.17
3.	**0.52**	0.06
4.	0.13	**0.62**
5.	**0.50**	0.09
6.	0.03	**0.67**
7.	**0.72**	−0.17
8.	**0.47**	0.07
9.	0.02	**0.71**
10.	−0.09	**0.81**
11.	0.06	**0.46**
12.	0.01	**0.75**
13.	**0.52**	−0.13
14.	**0.52**	0.19
15.	**0.71**	−0.07
16.	**0.39**	−0.05
17.	**0.32**	−0.16
18.	**0.66**	0.03

**Table 3:** Descriptive statistics for each EAS item, EAS subscale, and total scale scores.

Item	Mean	SD	Kurtosis	Skewness
1	4.21	2.08	−1.29	−0.05
2	3.76	1.99	−1.13	0.15
3	2.77	1.88	−0.57	0.78
4	6.20	1.30	3.97	−1.96
5	3.55	1.95	−1.01	0.18
6	6.26	1.38	4.86	−2.25
7	3.03	2.02	−0.81	0.69
8	2.78	1.88	−0.56	0.78
9	5.89	1.64	1.61	−1.58
10	5.98	1.54	2.75	−1.82
11	5.17	1.76	0.07	−0.91
12	5.87	1.58	2.32	−1.65
13	3.16	2.09	−1.09	0.51
14	3.87	2.09	−1.25	0.03
15	2.76	1.98	−0.48	0.90
16	3.35	2.52	−1.51	0.47
17	2.70	2.09	−0.61	0.92
18	2.28	1.92	0.79	1.44
AC	38.24	13.56	0.27	0.41
EP	35.37	6.20	2.28	−1.39
EAS	73.61	15.42	0.82	0.16

Note. AC, Anxious Clinging; EP, Experience Prolonging; EAS, Experiential Approach Scale.

**Table 4:** Psychological inflexibility, anxious clinging, emotional intelligence, and mental health symptoms in students aged 9–14.

	Female (*n* = 58) *M* (*SD*)	Male (*n* = 71) *M* (*SD*)	Total (*N* = 129) *M* (*SD*)	Cronbach’s α (95% CI)
Age	11.21 (1.36)	11.11 (1.28)	11.16 (1.32)	
AFQ-Y17	27.02 (9.83)	28.03 (13.05)	27.58 (11.68)	0.811 (0.768–0.847)
EAS*_Anxious Clinging_*	37.53 (12.34)	38.81 (14.54)	38.24 (13.56)	0.797 (0.712–0.846)
EQi-YV	113.15 (11.98)	114.56 (15.33)	113.97 (14.00)	0.910 (0.874–0.929)
RCADS-30	31.09 (11.38)	32.06 (13.65)	31.62 (12.64)	0.874 (0.835–0.903)
*Major depression*	3.09 (2.10)	3.54 (2.60)	3.33 (2.39)	0.647 (0.475–0.751)
*Panic disorder*	2.66 (2.61)	2.75 (3.03)	2.71 (2.84)	0.763 (0.673–0.824)
*Social Phobia*	5.03 (2.64)	5.97 (3.49)	5.55 (3.16)	0.658 (0.525–0.750)
*Separation anxiety*	4.72 (3.26)	3.92 (3.61)	4.28 (3.46)	0.743 (0.661–0.801)
*Generalized anxiety*	9.62 (2.81)	9.51 (3.16)	9.56 (3.00)	0.671 (0.548–0.746)
*Obsessive compuls.*	5.97 (2.62)	6.38 (3.29)	6.19 (3.00)	0.531 (0.355–0.652)

AFQ-Y17: Avoidance and fusion questionnaire; EAS: Experiential approach scale; EQi-YV: Emotional quotient inventory, youth version; RCADS-30: Revised child anxiety and depression scale.

**Table 5:** Correlations amongst psychological inflexibility, anxious clinging, emotional intelligence and mental health symptoms (N = 129).

	1. AFQ-Y17	2. EAS*_Anxious Clinging_*	3. EQi-YV
1. AFQ-Y17			
2. EAS*_Anxious Clinging_*	0.360 *		
3. EQi-YV	−0.174	−0.045	
4. RCADS-30	0.538 *	0.533 *	−0.180
*Major depression*	0.408 *	0.429 *	−0.436 *
*Panic disorder*	0.424 *	0.500 *	−0.059
*Social Phobia*	0.354 *	0.441 *	−0.154
*Separation anxiety*	0.339 *	0.251 *	−0.135
*Generalized anxiety*	0.278 *	0.337 *	0.006
*Obsessive compulsive*	0.499 *	0.333 *	−0.029

Note: AFQ-Y17, Avoidance and fusion questionnaire; EAS, Experiential approach scale; EQi-YV, Emotional quotient inventory; RCADS-30, Revised child anxiety and depression scale. * *p* < 0.01.

**Table 6:** Hierarchical regression predicting overall mental health symptoms (RCADS-30 total score), and major depression, panic disorder, social phobia, separation anxiety, generalized anxiety, and obsessive compulsive symptoms from Psychological Inflexibility (AFQ-Y17) and Anxious Clinging (EAS subscale).

	B	SE	β	*t*	*F*	*R^2^*	∆*R*^2^
** Dependent variable: RCADS-30**							
Step 1					48.625 ***	0.279	-
AFQ-Y17	0.571	0.082	0.528	6.947 ***			
Step 2					43.808 ***	0.405	0.135
AFQ-Y17	0.418	0.080	0.386	5.235 ***			
EAS*_Anxious Clinging_*	0.366	0.068	0.395	5.354 ***			
**Dependent variable: Major depression**							
Step 1					26.378 ***	0.191	-
EQi-YV	−0.074	0.014	−0.437	−5.136 ***			
Step 2					23.210 ***	0.295	0.104
EQi-YV	−0.065	0.014	−0.380	−4.697 ***			
AFQ-Y17	0.067	0.017	0.328	4.051 ***			
Step 3					23.193 ***	387	0.093
EQi-YV	−0.066	0.013	−0.386	−5.091 ***			
AFQ-Y17	0.043	0.017	0.210	2.594 *			
EAS*_Anxious Clinging_*	0.057	0.014	0.326	4.077 ***			
**Dependent variable: Panic disorder**							
Step 1					25.770 ***	0.171	-
AFQ-Y17	0.101	0.020	0.413	5.076 ***			
Step 2					28.209 ***	0.313	0.142
AFQ-Y17	0.065	0.019	0.268	3.360 **			
EAS*_Anxious Clinging_*	0.084	0.017	0.404	5.058 ***			
**Dependent variable: Social phobia**							
Step 1					17.632 ***	0.124	-
AFQ-Y17	0.096	0.023	0.352	4.199 **			
Step 2					19.284 ***	0.237	0.114
AFQ-Y17	0.061	0.023	0.222	2.635 **			
EAS*_Anxious Clinging_*	0.085	0.020	0.361	4.298 ***			
**Dependent variable: Separation anxiety**							
Step 1					15.111 ***	0.108	-
AFQ-Y17	0.098	0.025	0.328	3.887 ***			
Step 2					9.119 ***	0.128	0.020
AFQ-Y17	0.082	0.027	0.273	3.041 **			
EAS*_Anxious Clinging_*	0.039	0.023	0.153	1.702			
**Dependent variable: Generalized anxiety**							
Step 1					9.886 **	0.073	-
AFQ-Y17	0.070	0.022	0.271	3.144 **			
Step 2					10.022 ***	0.139	0.066
AFQ-Y17	0.044	0.023	0.172	1.922			
EAS*_Anxious Clinging_*	6.701	2.779	0.275	3.080 **			
**Dependent variable: Obsessive compulsive**							
Step 1					39.403 ***	0.240	-
AFQ-Y17	0.125	0.020	0.490	6.277 ***			
Step 2					22.681 ***	0.268	0.028
AFQ-Y17	0.109	0.021	0.425	5.157 ***			
EAS*_Anxious Clinging_*	0.039	0.018	0.180	2.184 *			

* *p* < 0.05; ** *p* < 0.01; *** *p* < 0.001.

## Data Availability

The data presented in this study are available on request from the corresponding author. The data are not publicly available due to privacy reasons.
